# Upregulation of microRNAs correlates with downregulation of HERV-K expression in Parkinson’s disease

**DOI:** 10.1007/s13365-024-01234-7

**Published:** 2024-10-18

**Authors:** Elena Rita Simula, Somaye Jasemi, Kay Paulus, Leonardo Antonio Sechi

**Affiliations:** 1 Department of Biomedical Sciences, Division of Microbiology and Virology, University of Sassari, Sassari, Italy; 2 Servizio di neuroabilitazione, Azienda Ospedaliera Universitaria Sassari, Sassari, Italy; 3 Struttura Complessa Microbiologia e Virologia, Azienda Ospedaliera Universitaria Sassari, Sassari, Italy

**Keywords:** Parkinson’s disease, HERVs, HERV-K, microRNAs

## Abstract

Human endogenous retroviruses (HERVs) involvement in neurological diseases has been extensively documented, although the etiology of HERV reactivation remains unclear. MicroRNAs represent one of the potential regulatory mechanisms of HERV reactivation. We identified fourteen microRNAs predicted to bind the HERV-K transcript, and subsequently analyzed for their gene expression levels alongside those of HERV-K. We documented an increased expression of four microRNAs in patients with Parkinson’s disease compared to healthy controls, which correlated with a downregulation of HERV-K transcripts. We hypothesize that specific microRNAs may bind to HERV-K transcripts, leading to its downregulation.

## Introduction

Parkinson’s Disease (PD) is characterized by the degeneration of dopaminergic neurons, particularly in the ventrolateral tier of the substantia nigra pars compacta.(Ramesh and Arachchige 2023) A key feature of PD is the presence of Lewy bodies, primarily composed of α-synuclein aggregates, in neurons and glial cells.(Calabresi et al. 2023) The neuronal damage leads to the depletion of dopamine causing severe motor symptoms such as rest tremors, rigidity, and bradykinesia, (Radad et al. 2023) as well as non-motor symptoms like cognitive impairment and neurobehavioral disorders.(Beitz 2014) Currently, there are no treatments to slow the disease’s progression, and available treatments focus exclusively on dampening motor symptoms related to dopamine deficiency.(Isaacson et al. 2023).

Like most neurodegenerative diseases, PD is multifactorial, and its cause remains unknown.(Müller-Nedebock et al. 2023) Recently, the involvement of endogenous human retroviruses (HERVs) in neurodegenation has been documented. HERVs make up approximately 8% of the human genome. They have been pinpointed as initiators or exacerbators of neurodegenerative processes, particularly in conditions like multiple sclerosis (MS) (Arru et al. 2020) and amyotrophic lateral sclerosis (ALS), (Arru et al. 2021; Simula et al. 2021; Garcia-Montojo et al. 2024) prompting the design of clinical trials aimed at targeting HERVs.(Ryan 2011; Diebold and Derfuss 2019).

While the association of HERVs and PD needs further investigation, the involvement of microRNAs (miRNAs) in the pathological mechanisms is well-established.(Li et al. 2021) MiR-330 is upregulated in PD patients playing a crucial role in modulating inflammatory responses by blocking key inflammatory pathways and repressing the polarization of macrophages to the M1 phenotype.(Feng et al. 2021) MiR-150, which is downregulated in PD patients, inhibits the production and release of proinflammatory cytokines, potentially contributing to the exacerbation of neuronal damage and disease progression.(Li et al. 2020).

In the present study, we investigated the gene expression levels of four specific miRNAs previously linked to neurodegenerative conditions. Through in silico analysis, the selected miRNAs were predicted to have binding affinity for the HERV-K transcript. To explore this potential interaction, we conducted a comprehensive analysis correlating the expression levels of miRNAs with key HERV-K genes, including -gag, -env, and -pol.

## Materials and methods

The study protocol received approval from the institutional review board at Sassari University Hospital (Azienda Ospedaliero-Universitaria, Sassari, Italy; IRB number 291123). Written informed consent was obtained from all participants.

### Samples

Whole blood samples were collected from patients diagnosed with PD and from blood donors in optimal health conditions. A total of 15 PD patients were recruited between January and October 2021 (6 females and 9 males; median age = 61 years) from the Neurorehabilitation Service, Azienda Ospedaliera Universitaria.

Fifteen blood donor samples were collected throughout the year 2021 at the Blood Transfusion Centre of Sassari (6 females and 9 males; median age = 63.5 years).

### Blood samples collection

Peripheral venous blood samples were obtained from the participants using K2-EDTA tubes. The collected whole blood was layered over an equal volume of Ficoll (Sigma-Aldrich, St. Louis, MO, USA) in a 15 mL tube and centrifuged for 20 min at 1800 RPM without brake. The peripheral blood mononuclear cells (PBMCs) were collected and stored at -80 °C in FBS with 10% DMSO for miRNA extraction.

### Identification of miRNAs able to bind HERV-K consensus sequence

The identification of microRNAs capable of binding to the consensus sequence of HERV-K [26] was performed using the online software miRDB. MiRDB is an online database designed for predicting miRNA targets and providing functional annotations. The targets within miRDB are predicted using a bioinformatics tool called MirTarget, which has been developed by analyzing thousands of miRNA-target interactions obtained from high-throughput sequencing experiments. Machine learning techniques have been employed using identified common features associated with miRNA binding and target downregulation to predict miRNA targets. Table 1 displays the list of selected miRNAs.

**Table 1:** Primers sequences. Table 1 shows the primer sequences used in the study, miRNAs marked with an asterisk (*) are those found to be upregulated in our study

Accession Number	Name	Sequence 5’– 3’	HERV-K Binding Site
*MIMAT0004488*	*hsa-miR-15a	CAGGCCAUAUUGUGCUGCCUCA	LTR, PRO, ENV
*MIMAT0004495*	*hsa-miR-22	AGUUCUUCAGUGGCAAGCUUUA	GAG, ENV LTR
*MIMAT0000259*	*hsa-miR-182	UUUGGCAAUGGUAGAACUCACACU	POL, ENV
*MIMAT0004613*	*hsa-miR-188	CUCCCACAUGCAGGGUUUGCA	LTR, POL
*MIMAT0004489*	hsa-miR-16	CCAGUAUUAACUGUGCUGCUGA	LTR, GAG, POL
*MIMAT0004499*	hsa-miR-26	CCUAUUCUUGGUUACUUGCACG	PRO, POL
*MIMAT0000451*	hsa-miR-150	UCUCCCAACCCUUGUACCAGUG	GAG, POL
*MIMAT0004558*	hsa-miR-181	ACCACUGACCGUUGACUGUACC	PRO, POL
*MIMAT0004543*	hsa-miR-192	CUGCCAAUUCCAUAGGUCACAG	ENV
*MIMAT0004614*	hsa-miR-193	UGGGUCUUUGCGGGCGAGAUGA	GAG
*MIMAT0000278*	hsa-miR-221	AGCUACAUUGUCUGCUGGGUUUC	GAG, ENV
*MIMAT0004683*	hsa-miR-362	AACACACCUAUUCAAGGAUUCA	LTR, PRO
*MIMAT0002873*	hsa-miR-502	AUCCUUGCUAUCUGGGUGCUA	GAG, PRO, POL, ENV
*MIMAT0004797*	hsa-miR-582-3p	UAACUGGUUGAACAACUGAACC	GAG, POL, ENV
*X59362*	U6	N/A– QIAGEN YP02119464	
*MIMAT0000101*	has-miR-103a-3p	AGCAGCAUUGUACAGGGCUAUGA	
*XM_047445854*	HERV-K-env-FW HERV-K-env-RV	CTGAGGCAATTGCAGGAGTT GCTGTCTCTTCGGAGCTGTT	
*NM_000194*	HPRT1-FW HPRT1-RV	GCTATAAATTCTTTGCTGACCTGCTG AATTACTTTTATGTCCCCTGTTGACTGG	

### RNA isolation and RT-qPCR analysis

Total RNA from PBMCs was purified using the miRNeasy Mini Kit (QIAGEN, Milano, Italy) following the manufacturer’s instructions and treated with DNase to remove DNA contamination with the Turbo DNA-free kit (Thermofisher), following the manufacturer’s instructions. Then, the RNA concentration was measured with the Nanodrop (Thermofisher) and all the samples were adjusted to the same concentration. First-strand cDNA synthesis was performed using QuantiTect Reverse Transcription Kit (QIAGEN, Milano, Italy). No-RT (no Reverse Transcriptase) for each sample and no-TC (no Template Control) controls were made to check for DNA and reagent contamination, respectively. Subsequently, samples were analyzed by Real-Time-PCR using Quantinova SYBR Green PCR kit (QIAGEN, Milano, Italy), following the manufacturer’s instructions.

The relative mRNA expression levels were calculated by the 2^−∆∆Ct^ method and HPRT1 mRNA levels were used for normalization. Gene-specific primer pairs are listed in Table S1.

### miRNA isolation and RT-qPCR analysis

miRNAs from PBMCs were purified using the miRNeasy Mini Kit (QIAGEN, Milano, Italy) and treated with DNAse to remove DNA contamination with the Turbo DNA-free kit (Thermofisher), following the manufacturer’s instructions. Then, the RNA concentration was measured with Nanodrop (Thermofisher) and all the samples were adjusted to the same concentration. miRNAs were reverse transcribed using miRCURY LNA RT Kit (QIAGEN, Milano, Italy). No-RT (no Reverse Transcriptase) for each sample and no-TC (no Template Control) controls were made to check for DNA and reagent contamination, respectively. Subsequently, samples were analyzed by Real-Time-PCR using miRCURY LNA SYBR Green PCR Kit (QIAGEN, Milano, Italy) following the manufacturer’s instructions. Relative levels of miRNAs were calculated by the 2^−∆∆Ct^ method and the average of the reference genes, snRNA-U6, and miR-103a-3p expression levels were used for normalization. Gene-specific primer pairs are listed in Table S1.

### Statistical analysis

Data was analyzed with GraphPad Prism version 8 (GraphPad). The Kolmogorov-Smirnov test of normality was applied to all data sets to determine whether a data set was normally distributed. Differences between means were assessed by unpaired two-sided Student’s t-test followed by Dunnett’s post-hoc testing. For the comparison of groups that did not demonstrate normal distribution or were heteroscedastic, the non-parametric tests Mann-Whitney with correction for multiple comparisons were used. Values in graphs are expressed as mean ± standard error mean (SEM) for parametric comparisons and as median with interquartile range (IQR) for non-parametric tests. The correlation of two variables was assessed by the Spearman R test. Statistical significance was achieved when *p*-value < 0.05.

## Results

We investigated the expression levels of HERV-K-env and specific microRNAs (miRNAs) in both Parkinson’s disease (PD) patients and healthy controls (HCs).

**Fig. 1:**
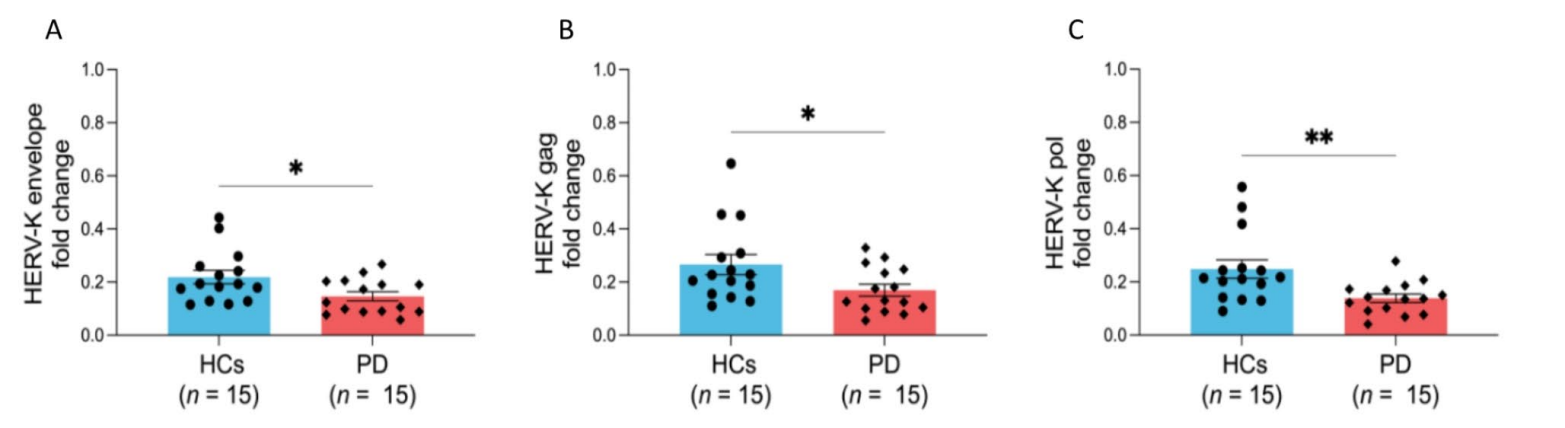
HERV-K gene expression levels. Gene expression levels of HERV-K-env (**A**), HERV-K-gag (**B**), and HERV-K-pol (**C**) in HCs and PD subjects. A single asterisk (*) indicate *p* < 0.05, and double asterisks (*) indicate *p* < 0.01, based on Fisher’s exact test

Our analysis revealed a significant downregulation of the HERV-K-env transcript in PD patients compared to the healthy control group (Fig. 1). Specifically, we observed a reduction in the expression levels of HERV-K, with statistically significant differences noted for -env (*p* = 0.01) (Fig. 1A), -gag (*p* = 0.02) (Fig. 1B), and -pol (*p* = 0.002) (Fig. 1C) suggesting a potential link between the altered expression of HERV-K genes and the pathophysiology of PD.

**Fig. 2:**
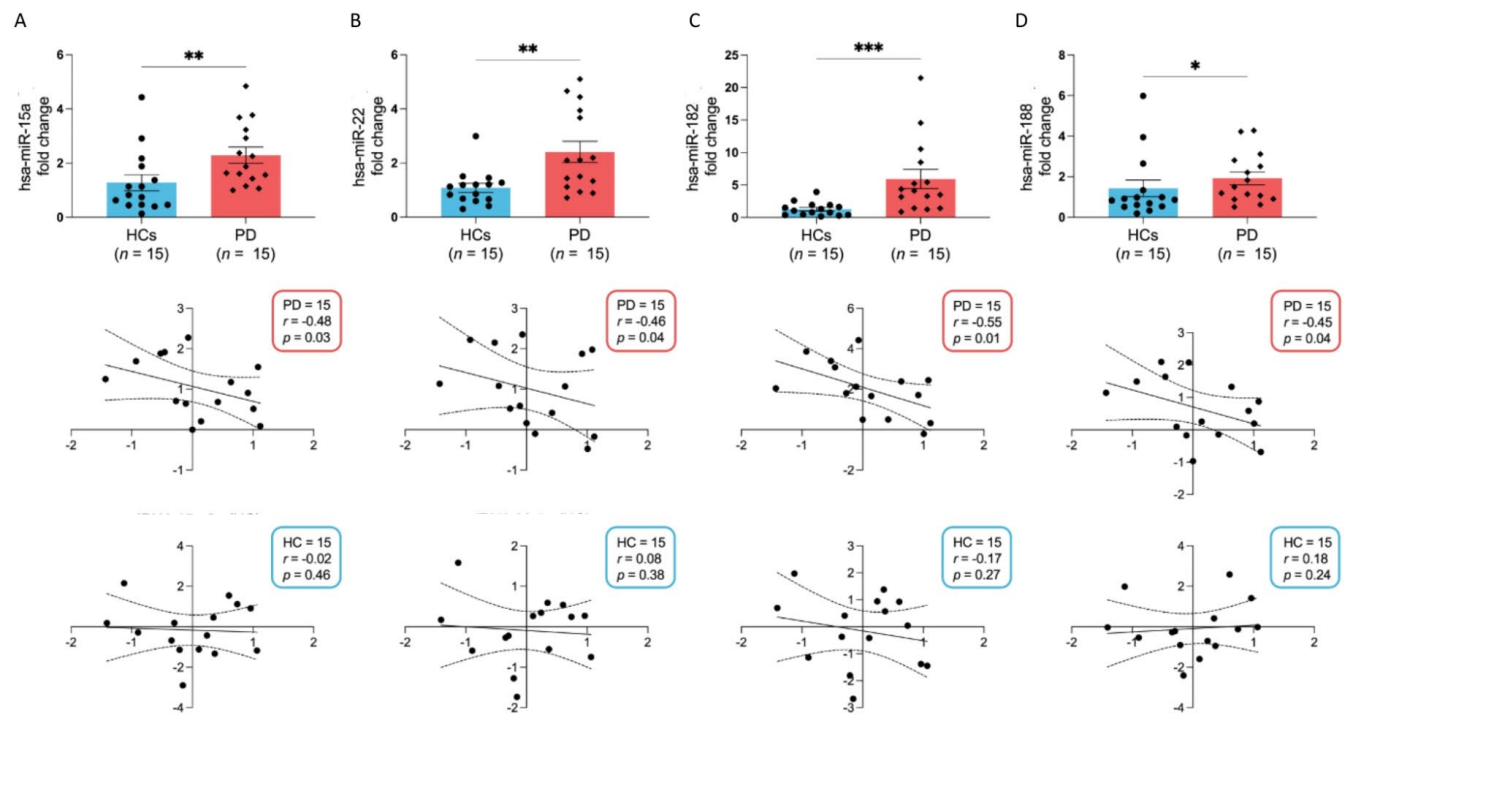
Gene expression levels of miRNAs and their correlation with HERV-K-env gene expression levels. Gene expression levels of miRNAs (upper section) miR-15a (**A**), miR-22 (**B**), miR-182 (**C**), and miR-188 (**D**) and their correlation with HERV-K-env gene expression levels in PD (middle section) and HCs individuals (bottom section). A single asterisk (*) indicate *p* < 0.05, double asterisks (*) indicate *p* < 0.01, and triple asterisks (***) indicate *p* < 0.001, based on Fisher’s exact test

Regarding the expression levels of the miRNAs investigated, four miRNAs among the fourteen previously identified to bind HERV-K transcripts were found to be significantly upregulated in PD patients compared to healthy controls. The specific miRNAs and their respective *p*-values were as follows: miR-15a (*p* = 0.0029), miR-22 (*p* = 0.0021), miR-182 (*p* = 0.0002), and miR-188 (*p* = 0.03). Furthermore, the expression levels of these miRNAs showed a negative correlation with those of HERV-K-env, indicating a potentially linked mechanism wherein the elevated miRNA levels may contribute to the downregulated expression of HERV-K-env. The correlations were statistically significant and detailed as follows: HERV-K-env with miR-15a (*r* = -0.48, *p* = 0.03), miR-22 (*r* = -0.46, *p* = 0.04), miR-182 (*r* =-0.55, *p* = 0.01), and miR-188 (*r* = -0.45, *p* = 0.04). This negative correlation was observed exclusively in the PD population and was not present in the healthy control (HC) subjects. (Fig. 2)

## Discussion

During the late stages of PD, HERVs are deregulated in several critical brain regions and cell types, (Gordevičius et al. 2023) suggesting their potential role in the disease’s progression.

In addition, miRNAs strongly influence the activation and polarization processes of microglia (Li et al. 2021) which can adopt either a pro-inflammatory (M1) or anti-inflammatory (M2) phenotype.(Guo et al. 2022) For instance, miR-124 promotes the M2 phenotype by targeting components of the NF-κB signaling pathway. Through its upregulation is possible to shift microglia toward a neuroprotective state, thereby reducing inflammation and supporting neuronal survival.(Yao et al. 2019; Chen et al. 2021) Similarly, miR-155 is a well-documented promoter of the M1 phenotype. It enhances the production of pro-inflammatory mediators such as TNF-α and IL-6. Elevated levels of miR-155 have been observed in various neurodegenerative diseases, including AD and MS, indicating its role in exacerbating neuroinflammation.(Butovsky et al. 2015; Ma and Zhao 2023).

In the present study, we observed the upregulation of four specific miRNAs in PBMCs of PD patients compared with healthy subjects. MiR-15a, involved in various cellular processes such as apoptosis, cell cycle regulation, and inflammation, has been found elevated in the plasma of AD patients.(Bekris et al. 2013) The expression of miR-22 has been found significantly elevated in Parkinson’s patients and associated with abnormal levels of vascular inflammatory markers.(Yu et al. 2020) Inhibition of miR-182 has been shown to protect against experimental stroke in vivo and reduce astrocyte injury and inflammation in vitro by modulating cortactin activity.(Alhadidi et al. 2022) The dysregulation of miR-188-5p plays a role in the pathogenesis of AD by causing synaptic dysfunction and cognitive deficits linked to Aβ-mediated pathophysiology.(Lee et al. 2016).

For the first time, this study establishes a significant association between the investigated miRNAs and PD. Our findings reveal a robust negative correlation between the miRNAs’ gene expression levels and the HERV-K-env gene suggesting a potential regulatory mechanism where miRNAs may contribute to the downregulation of HERV-K-env in PD patients. Notably, the negative correlation is observed exclusively in the patient population and not in the controls. The upregulation of miRNAs in PD patients and their strong inverse relationship with HERV-K-env transcript levels highlight a targeted regulatory mechanism that might be activated or amplified in the disease context.

The downregulation of HERV-K has been linked with neural stem cell differentiation, critical for maintaining neural plasticity and regeneration, and essential for normal brain function and repair mechanisms.(Wang et al. 2020) It has been observed that the repression of HERV-K in neurons reduces the transcripts of interferon-sensitive genes.(Turelli et al. 2020) This suggests its role in modulating neuronal immune responses, which are crucial in PD, where inflammation and immune response contribute to the pathology (Tansey et al. 2022).

Based on these findings, we hypothesize the upregulation of miRNAs as a compensatory response to mitigate the expression of HERV-K, whose levels in the early stages of the disease are not yet known.

To address the limitations of our study, we plan to increase the number of subjects to obtain a more representative study population and conduct experiments on patients at different stages of PD to identify potential variations in HERVs and miRNAs gene expression levels. We will investigate the potential involvement of other HERV families, such as HERV-W and HERV-H with other miRNAs, to better understand the role of these retroviruses in the context of PD. Moreover, while our observed correlation is significant, it does not necessarily imply a causal relationship. Functional studies are required to elucidate the precise mechanisms by which miRNAs regulate HERV-K expression.

Despite the preliminary nature of our study, we highlight the involvement of miRNAs in PD, potentially correlating with the inhibition of HERV-K. Our findings underscore the need for further investigation to elucidate the underlying mechanisms and identify potential therapeutic targets in this emerging field.

## Data Availability

No datasets were generated or analysed during the current study.
